# A Case of Purpura Hemorrhagica

**Published:** 1846-05

**Authors:** W. D. Harris

**Affiliations:** Utica, Mississippi


**Art. V.**—*A Case of Purpura Hemorrhagica.* By W. D. Harris, M. D., of Utica, Mississippi.

The following case which occurred in my practice being of a somewhat novel character may not be uninteresting, I hope, to some of the numerous readers of the Journal.

October 9th, 1845. I was called to visit Miss M. S., aged 23 years, of small size, dark hair and complexion, health and constitution delicate from childhood, whom I had attended sixteen months previous for spinal irritation, together with a slight affection of the uterus, and considerable bronchial difficulty, with pain in the left side, &c.

When I arrived on this occasion, I found her laboring under the following symptoms: hemorrhage from the nose, somewhat profuse, of 30 hours duration—also from every part of the body, as well as the head, neck, and face; the blood oozed out through the pores of the skin, resembling mosquito bites, in some places in clusters thicker than others. Knowing her constitution to be delicate, I supposed that there must be some derangement of the catamenia; but on inquiry found that her health in that respect was as good as usual. Previous to my visit, she had been under the care of a talented quack for a pain in the side, with some debility of the lungs, for which she was using externally veratria ointment, and taking internally deutiodide of mercury, with iodide of potassium, up to the time when I made my first visit; I prescribed alum water to the nostrils and a plug saturated with the same to be inserted into the nose; wash for the body, dilute acid of vitriol; gave a blue pill and sulph. quinine sufficient to move the bowels; spirits of nitre and paregoric as a diuretic in teaspoonful doses every three hours, and Dover's powder sufficient to allay nervous excitability, and procure rest.

10th. Saw my patient at 9 A.M.; found her relieved from the hemorrhage of the skin, but from the left nostril blood still continued to flow freely. Medicine had moved the bowels; tongue clean; pulse 100. The dilute oil of vitriol had a beneficial effect on the skin, though attended in its application with some nervous excitement, which was allayed by Dover's powder.

As I looked on the hemorrhage as passive from the beginning, I prescribed dilute creosote to the nose with a plug; oak-bark and alum wash to the skin, and gave sulph. quinine in 3 grain doses alternarely with Dover's powder in 5 grain doses every four hours, and persesquinitrate of iron in 12 drops three times a day; kept the bowels open with mild cathartics.

11th. Found my patient still bleeding at the nose, though less profusely; pulse feeble, beating 110 per minute; skin soft; patient had spent a restless night, attended with strong hysterical symptoms, and slight derangement of intellect at times. Continued the creosote to the nose, finding it to be the best among the many remedies that were tried. Ordered the alum hip-bath; continued the quinine and Dover's powder, and gave 10 drops of tinct. iodide of iron instead of the nitrate. Her mouth now was slightly touched with the mercury, either the blue pill, or the stronger preparations which she had been taking before my first visit.

12th. Saw her this morning in consultation with my friend, Dr. Bush; found her very much as on the day previous; continued the same treatment, adding the compound tinct. of catechu to be applied to the nose and given internally.

13th. Still bleeding at the nose, though much less profusely; continued the creosote, as it seemed to be the only application which was of any service; symptoms much the same as yesterday; hysteria still continues; treatment the same, only syrup morphine added to control the nervous irritability.

14th. This morning found my patient relieved from hemorrhage from the nose; pulse small and frequent; skin moist; extremities cool; exceedingly feeble and easily excited to crying or laughter; mouth sore, and cheeks swollen, notwithstanding the bowels have been opened every day with mild cathartics. This day the catamenia came on, and at times was somewhat profuse, producing the greatest alarm, but all persuasions of her mother or myself were unavailing to induce her to suffer any local application to be made.— Continued general treatment; added tinct. myrrh in drachm doses. She now began to improve slowly, and was furnished with such medicines from the shop as her condition required from time to time.

Dec. 3. Was called to my patient again; on arriving I found her laboring under uterine hemorrhage in the strict sense of the word. This of course produced much alarm. I explained the nature of the case, and assured her that there was no danger, as it was of very frequent occurrence though not in unmarried women. It readily yielded to the usual treatment; since which time she has recovered her usual health and spirits.

I might mention in conclusion that this young lady belongs to one of the best families in the farming community, who never knew want, or participated in luxurious indulgences. I present the case as it occurred without comment. How far the powerful agents she was using had any share in producing the state of things above recited I shall not attempt to determine. The pathology of purpura is still involved in great obscurity. The multiplication of cases must ultimately clear it up. In that view, I trust the one which I have given may be found to be of some value.

March 10th, 1846.

